# Side-chain Liquid Crystal Polymers (SCLCP): Methods and Materials. An Overview

**DOI:** 10.3390/ma2010095

**Published:** 2009-03-11

**Authors:** Tomasz Ganicz, Włodzimierz Stańczyk

**Affiliations:** Centre of Molecular and Macromolecular Studies, Polish Academy of Science, Sienkiewicza 112, 90-363 Łódź, Poland; E-Mail: was@cbmm.lodz.pl

**Keywords:** Liquid crystals, side chain polymers, polymer modification, polymer synthesis, self-assembly

## Abstract

This review focuses on recent developments in the chemistry of side chain liquid crystal polymers. It concentrates on current trends in synthetic methods and novel, well defined structures, supramolecular arrangements, properties, and applications. The review covers literature published in this century, apart from some areas, such as dendritic and elastomeric systems, which have been recently reviewed.

## 1. Introduction

**Figure 1:**
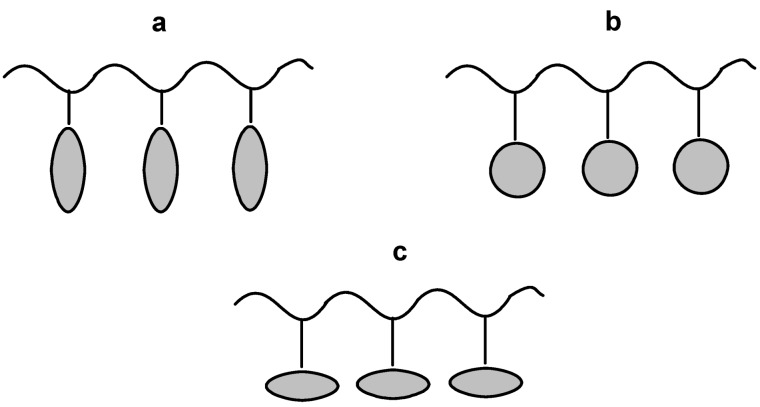
Possible architectures of SCLCP’s; (a) rod-like terminal, (b) disc-like, (c) rod-like lateral.

Over thirty years ago the work of Finkelmann and Ringsdorf [1,2] gave important momentum to synthesis of side chain liquid crystal polymers (SCLCPs), materials which combine the anisotropy of liquid crystalline mesogens with the mechanical properties of polymers. Although there were some earlier attempts [2], their approach of decoupling the motions of a polymer main chain from a mesogen, thus allowed side chain moieties to build up long range ordering (Figure 1).

They were able to synthesize polymers with nematic, smectic and cholesteric phases *via* free radical polymerization of methacryloyl type monomers [3]. Systems possessing discotic type mesogens, able to generate various columnar phases have also been reported [4]. The decoupling (flexible) spacer concept opened the way to the whole variety of SCLCP materials with a wide range of structural design and synthetic methods, which in many cases underwent a dramatic change. The progress of fundamental research over the years was driven by applied studies devoted to sensing and memory storage devices, transistors, PLED’s and polymer networks [5,6]. 

The aim of this paper is to update recent results in the synthesis of novel SCLCPs. Some earlier reviews devoted to selected aspects of chemistry and properties of the LC side chain polymers [7,8,9] have already appeared, however they were focused on more narrow areas. Related systems, e.g. dendrimers [10] and elastomers [11] have been reviewed very recently (2007), therefore our overview does not cover these areas of research.

## 2. Synthetic methods

In the past, majority of side chain liquid crystal materials were made by two synthetic routes – free radical polymerization of acrylic type monomers, bearing mesogenic moieties [12,13] and hydrosilylation of mesogenic terminal alkenes with linear poly[(methylhydro)siloxanes], copolymers bearing alkylhydrosiloxane monomeric units [12,14] or polymer systems modified with reactive Si-H bonds [15]. These methods could be, in principle, applied only in synthesis of thermotropic SCLCP’s having different backbones, organic and rigid (acrylates) or purely inorganic, and flexible ones (polysiloxanes). The common feature of both types of SCLCPs, obtained by either of synthetic pathways, is high polydispersity index ( PDI = M_w_/M_n_ >2 ). On a molecular level it means that there is a limited possibility of exact tuning the polymerization degree (length of individual macromolecules) and thus the liquid crystalline properties of the synthesized materials. Such the problems can be avoided once conditions for living or controlled polymerization are created (see Section 2.3) or when liquid crystalline moieties are attached to star shaped or dendritic systems [9].

### 2.1. Free radical polymerization

**Figure 2:**
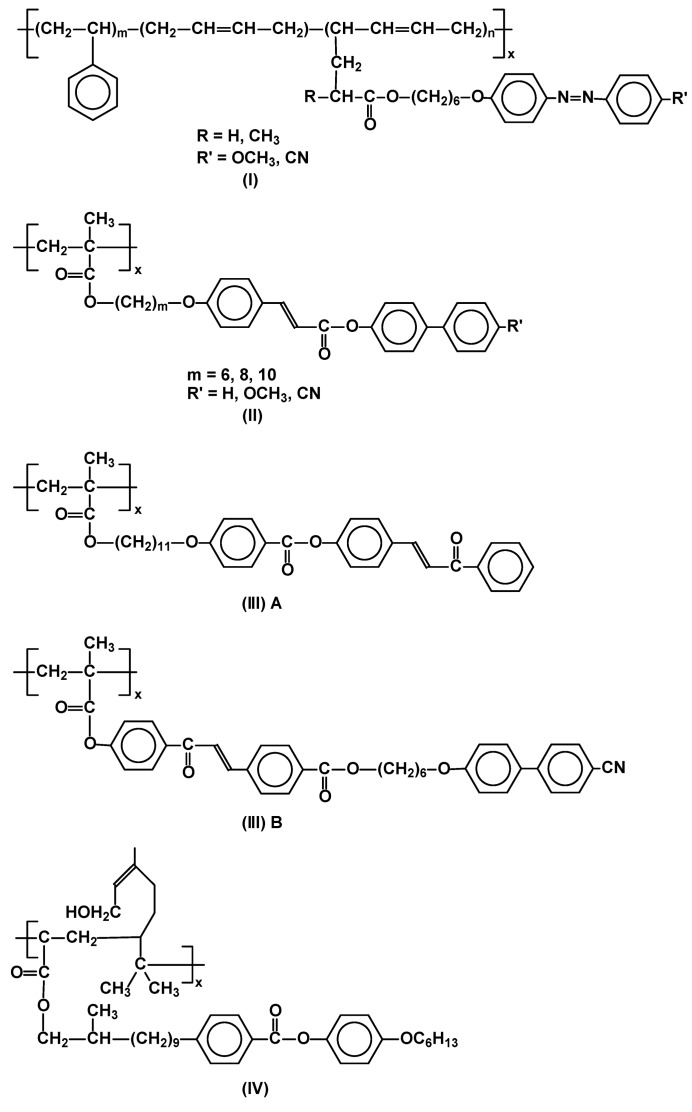
Functional SCLCP’s synthesized in the process of free radical polymerization.

Polymerization of acrylic monomers, in the presence of free radical initiators, is a relatively simple method and is often applied in synthesis of new and functional side chain systems anchored on carbon polymer backbone [16]. Azobenzene elastomers (styrene-butadiene-styrene) (**I**) for tunable gratings were prepared recently by this route [17] as well as functionalized polymer precursors for liquid crystalline polymer networks based on cinnamate (**II**) [18] and chalcone (**III**) [13] moieties in the side chain (Figure 2). The LC properties of polymers depend not only on the structure of the side chain mesogen or length of flexible spacer, but also on the flexibility of polymer skeleton and the latter is affected both by molecular weight (M_n_) and polydispersity (PDI = M_w_/M_n_) [15,19,20].

Thus, once properties of LC materials obtained by chain polymerizations are compared, one has to take into account not only their M_n_, but also the PDI value. Several novel, functionalized SCLCP's have been recently described including copolymers having poly(geraniol-*co*-MMA) main chain (**IV**) [20] (Figure 2) and analogous copolymers of MMA and terpineol [21], and limonene [22]. Their properties were compared with poly-methacrylates and -acrylates bearing the same phenyl benzoate mesogenic group, though they were vaguely characterized. It has been suggested that the nature of the new backbone containing terpenoid moiety allows for enhanced thermal stability of mesophase. As expected, the materials were highly polydisperse (PDI ~3.5).

An interesting synthetic approach was applied for the first stage of SCLCP’s synthesis. A very effective enzymatic method was used in transesterification, leading to acrylic monomers with methoxybiphenyl mesogenic pendants. After 30 min., 100% conversion was found at 30 °C in acetone, in the presence of Novozyme 435 (an immobilized lipase from *Candida antarctica*). Radical polymerization, which followed proved to be of much less success, leading to polymer with a rather low molecular weight of M_n_ = 7,000-10,000. The resulting material had showed a nematic phase in a very narrow range of temperature Cr 89 N 113 I [23]. 

### 2.2. Hydrosilylation and side chain LC polysiloxanes

Another traditional synthetic route leading to specific type of SCLCP’s with purely inorganic backbone – polysiloxanes is currently still being exploited. In this section, some other methods leading to polysiloxane side chain system shall be presented as well. Siloxane polymers are of significant technological interest. The highly flexible Si-O bonds provide materials with much lower T_g_ than liquid crystalline polyacrylates and polymethacrylates. At present the hydrosilylation reaction, catalyzed by platinum complexes, has been described for SCLC polysiloxanes capable of generating mesomorphic properties at moderate (25-45 °C) temperatures. Most of the current studies concentrate on synthesis of polymers generating chiral smectic C and cholesteric mesophases. 

**Figure 3:**
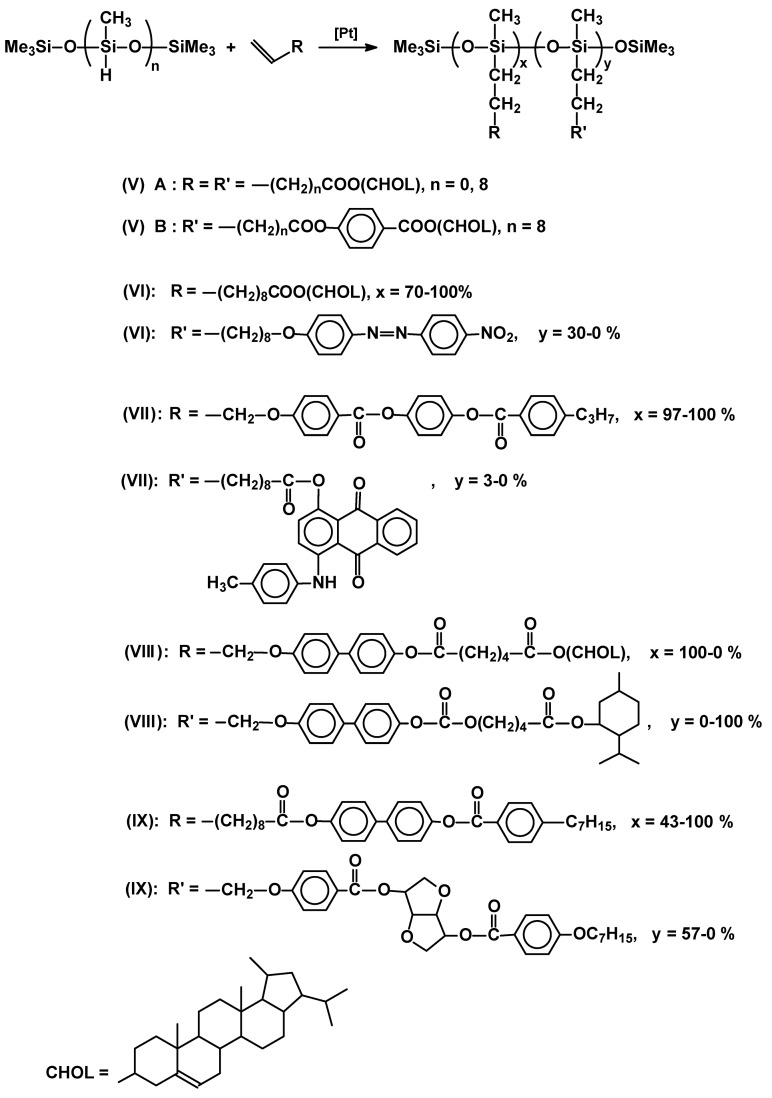
Structures of SCLCP polysiloxane copolymers obtained via hydrosilylation.

Although catalyzed hydrosilylation of mesogenic (or promesogenic) alkenes with Me_3_Si[OSi(Me)H]_n_OSiMe_3_ polymers leads to almost total addition of Si-H moiety across the alkene double bond, the polydispersity (PDI~2) is created at the stage of synthesis of the polysiloxane substrate. It is a good coincidence that some of the authors used the same “silicone” oligomeric substrate of M_n_ = 700-800, so the reader can directly compare the thermal properties of these materials having various mesomorphic side chains (Table 1). They vary by the structure of a flexible spacer between cholesteric group (**V**) [24] and siloxane backbone or form random copolymers by grafting different side chains. Side chain liquid crystalline polysiloxanes can be of potential value for optical applications, such as switches, filters, data storage systems. Thus, current research is also focused on copolymers bearing dichroic dyes, azobenzene (**VI**) [25] or anthraquinone (**VII**) [26] derivatives, copolymers containing cholesterol and menthol (**VIII**) [27] or a mesogen and non-mesogenic chiral systems (**IX**) [27] (Figure 3).

In the latter case it was possible to generate chiral smectic C phase in a system with two different side chains (one containing non-mesogenic chiral group). However mesomorphic properties deteriorated and disappeared once the molar content of chiral groups exceeded ~30%.

As the poly(methylsiloxane) backbone is the same in all cases it is easy to compare the effect of type of mesogens and their density along the backbone on thermal properties and stability of mesomorphic phase which determines their potential application. The importance of the length of the flexible spacer is clearly demonstrated (entry V, Table 1). The hydrosilylation reaction was also used extensively in the synthesis of highly branched star, comb-like dendritic systems [29,30], and polymers with increased density of side chain mesogens [31].

**Table 1:** Thermal properties of SCLC polysiloxanes of various structure, based on the same main chain (numbers refers to Figure 3).

Polymer	mesogen M [mol %]	n in (CH_2_)_n_ at M	mesogen M’ [mol %]	n’in (CH_2_)_n_ at M’	T_g­_ (T_m_) [°C]	T_i_ [°C]	ΔH [J/g]	Ref.
V	100	2	-	-	84	-	-	[24]
V	100	10	-	-	24	154	2.61	[24]
V	100	10	-	-	39	234	1.73	[24]
VI	100	10	-	3	16.5	154	1.93	[25]
VI	98	10	2	3	20.5	153	2.01	[25]
VI	90	10	10	3	16	141	2.04	[25]
VI	70	10	30	3	4	109	1.81	[25]
VII	100	3	-	10	47	273	1.24	[26]
VII	99.6	3	0.4	10	52	135	2.88	[26]
VII	97	3	3	10	61	129	0.48	[26]
VIII	100	3	0	3	46	192	4.52	[27]
VIII	40	3	60	3	32.5	110	1.36	[27]
VIII	0	3	100	3	54	77	0.27	[27]
IX	100	10	0	3	107	254	3.96	[28]
IX	86	10	14	3	100	211	2.50	[28]
IX	71	10	29	3	95	164	0.94	[28]
IX	57	10	43	3	76	92	-	[28]

**Figure 4:**
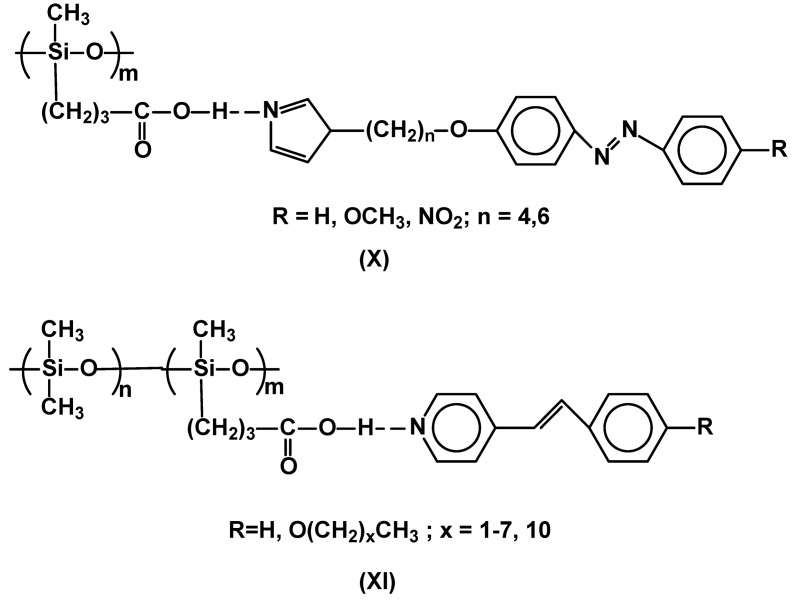
FLiquid crystalline polysiloxanes having hydrogen bonded side, calamitic moieties.

Interest in self-assembled liquid crystal polymers has expanded considerably in recent years. Syntheses of such supramolecular architectures largely increase the range of possible chemical structures and synthetic methods. The hydrogen bonded SCLCP’s (see also Section 3) based on polysiloxane chains have been presented. Two types of siloxane polymers and copolymers, the latter having up to 59 mol % of -Me_2_SiO-, monomeric units, were described. In both cases the LC properties were generated by interactions of 3-carboxypropyl side groups of polysiloxanes and imidazole ring in azobenzene (**X**) [32,33] or stilbazole derivatives (**XI**) [34] (Figure 4). Such strategy for construction of SCLCPs offers relative simplicity of their preparation and additional tuning of liquid crystalline properties by changing the ratio of hydrogen bonded species. On the other hand the synthesis of 3-carboxypropyl-substituted polysiloxanes from (3-cyanopropyl)methyldichlorosilane (and dimethy-dichlorosilane for copolymers) *via* hydrolysis and condensation leads to polymers with PDI > 2 [35].

### 2.3. Living and controlled polymerizations

Living polymerizations including anionic, cationic and ring opening metathesis processes require in practice that, the rate constant of propagation is much higher than those of termination and chain transfer. Quantitative and fast initiation leads to polymers of controlled molecular weight and polydispersity, as well as allows for efficient synthesis of block copolymers. Such processes were widely studied in the past for syntheses of SCLCP [36] and are still of some interest [37,38], mainly in synthesis of SCLC poly(oxetanes).

**Figure 5:**
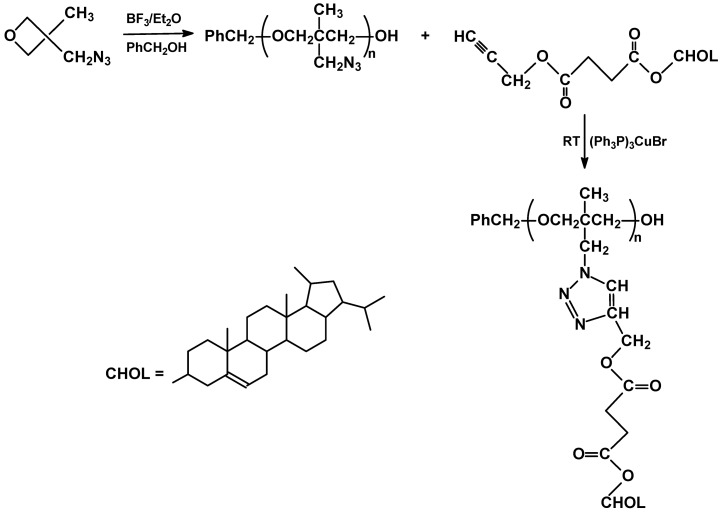
Ring opening polymerization and “click“ chemistry applied in synthesis of SCLCP's.

An interesting combination of cationic ring opening polymerization and “click” chemistry has been presented as a new promising synthetic route. BF_3_ catalyzed polymerization of 3-azidomethyl-3-methyloxetane led to a polymer backbone bearing side azide groups which then were coupled with propargyl monocholesterylsuccinate in the presence of Cu^I^ complex [39] (Figure 5).

**Figure 6:**
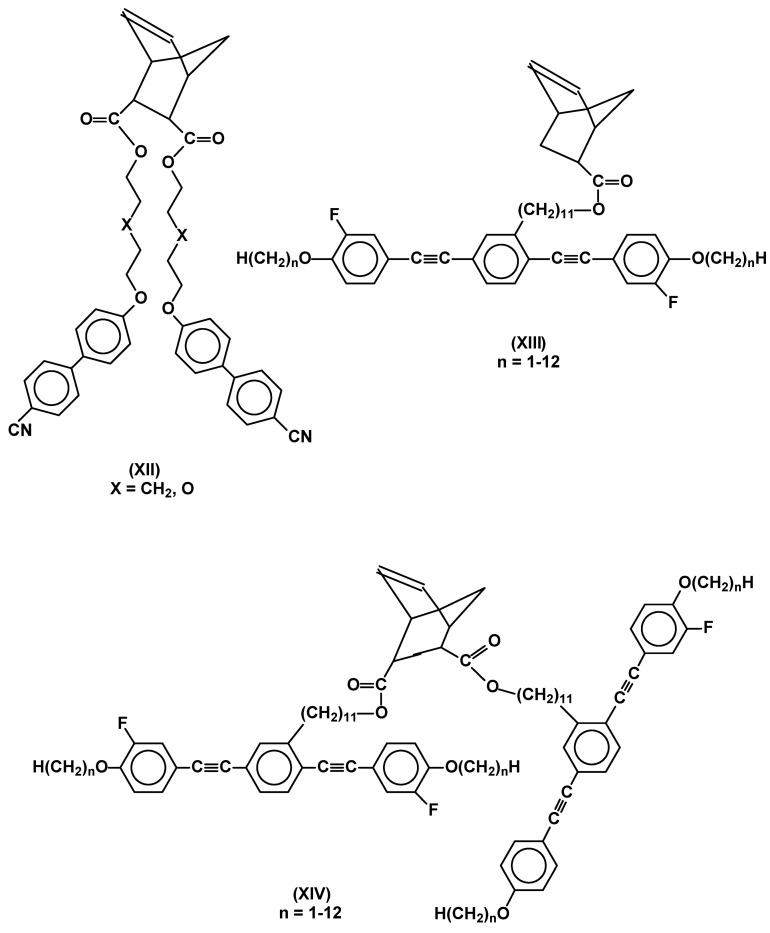
Examples of norbornene monomers used in ROMP.

Well defined polymers can be obtained from strained cyclic monomers such as norbornenes or some butenes, having mesomorphic substituents, by ring opening metathesis polymerization. Propagation is in such cases irreversible. The most often used initiators are carbene organometallic complexes of transition metals such as ruthenium, molybdenum and tungsten. Some limitations concerning complex stability, chain transfer, rate of initiation and functional groups in mesomorphic monomers have to be taken into account [40]. Both ruthenium and molybdenum complexes (Grubbs and Schrock type catalysts) were recently used in ROMP of substituted norbornene monomers (XII – XIV in Figure 6), bearing terminal or lateral rod-like substituents. Whereas, the two described molybdenum initiators proved to be effective [41,42], the ruthenium ones required additional stabilization exerted by sterically hindered and strongly electron donating N-heterocyclic carbene ligand [41]. Molybdenum initiators appear, in general, to be superior to the studied ruthenium compounds, both in terms of obtained molecular weight and polydispersity of polymers (PDI = 1.4-2.4 for [Ru]; PDI = 1.08-1.31 for [Mo]) [7].

However, in most cases all types of living polymerizations require ultra pure solvents, reagents and with few exceptions, exclusion of oxygen, and moisture. Many mesogenic moieties contain functional groups, which rule out living polymerization methods such as cationic, ionic and metathesis processes [40].

**Figure 7:**
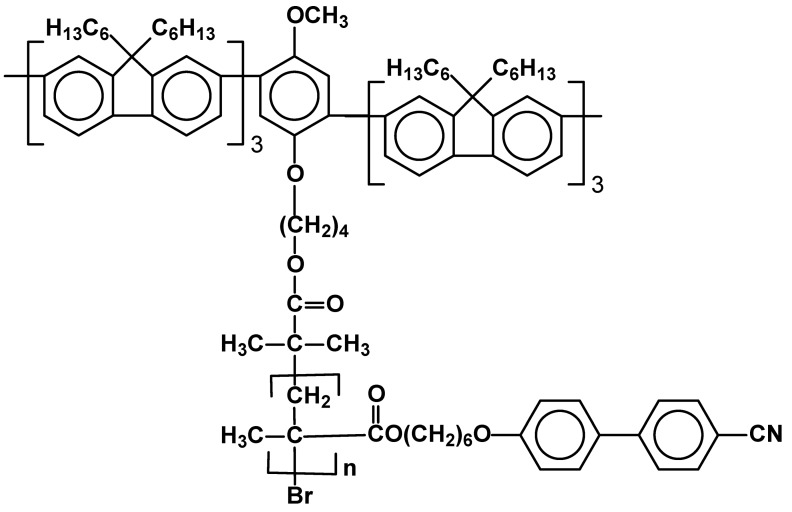
Structure of photoluminescent SCLCP obtained by in the controlled polymerization process.

The current synthetic interest is, thus, focused on controlled radical polymerization – atom transfer radical polymerization (ATRP) [43,44,45] and related reversible addition-fragmentation chain transfer technique (RAFT) [46]. Controlled polymerizations have been applied to synthesize well defined homopolymers with controlled molecular weight, block copolymers, star polymers, etc. [47,48]. A special type of so called mesogen-jacketed polymers have been recently highlighted [49]. The possibility of synthesis of SCLCPs, by ATRP, having different but narrowly distributed average molecular weight (PDI = 1.12-1.17) allows to study the effect of polymerization degree on thermal properties and extent of mesophase formation [40]. Block copolymers having segments containing monomeric units with liquid crystalline moiety attracted much attention. Current research concentrates mainly on functional materials [51,52] e.g. triblock [53,54] copolymers, made in the presence of difunctional initiators or/and macroinitiators based on polyethylene glycol or n-butane chain terminated with bromoisobutyrate. Mono(bromo)isobutyrate species were exploited in synthesis of a novel diblock photoluminescent copolymer (Figure 7) [55].

**Figure 8:**
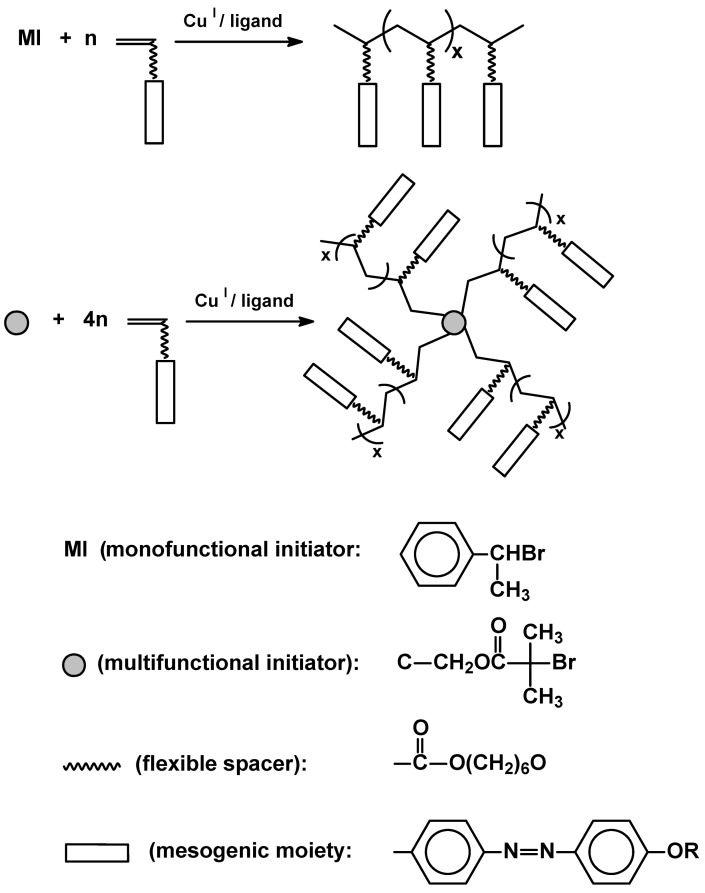
Linear and star-shape SCLCPs synthesized by ATRP.

A very special type of copolymer with a rather far reaching aim of generation of electronically addressed paper was reported. Successful synthesis involved ATRP of LC acrylate grafted from cellulose surface [56]. Once monofunctional initiator is replaced by a multifunctional one (Figure 8) star shape LC polymers can be obtained. Due to their spatial shape and rheological properties there is still interest in studying properties of many novel structures (both homo- [57,58] and copolymers [59]), and their application [47,48,60].

A more recently developed RAFT (Reversible Addition-Fragmentation Chain Transfer) technique has been also applied in synthesis of SCLCPs, utilizing 2-(2-cyanopropyl)ditihiobenzoate (CPDB) as the RAFT agent, which forms dormant species [61,62]. The same process was also applied in an elegant way to synthesize mesomorphic nanocomposite materials - σ-complexes of SCLCP with Ag nanoparticles surface via RAFT generated thiol group from S-benzyldithiobenzoate [63].

In recent years, the major development in controlled radical processes evidently shifted synthetic interest of novel SCLCP’s in the direction of ATRP and RAFT methods, while “traditional” ionic living polymerizations are not attracting much attention.

## 3. Self-assembled side chain liquid crystalline systems

**Figure 9:**
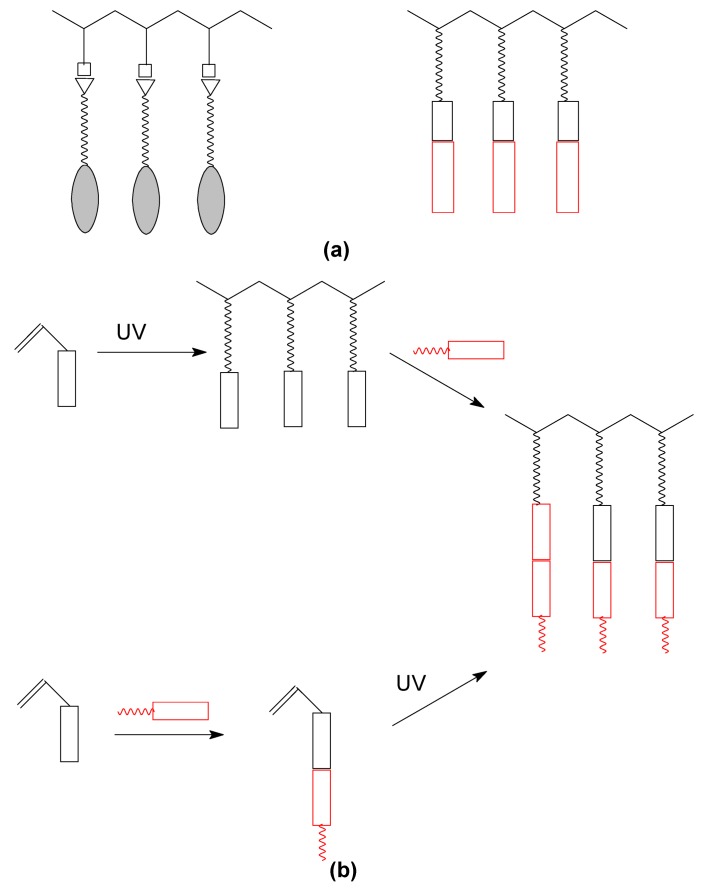
Self-assembled SCLCP’S. (a) Non-covalent bonding in flexible spacer or mesogenic group; (b) alternative synthetic pathways.

The knowledge of structure-properties relationship is crucial for obtaining LC materials with optimum desired properties. Apart from SCLCPs in which the side groups are covalently bonded to the polymer chain, the other pathway involves formation of LC polymers via self-assembly of relevant moieties, exploiting non-covalent interactions, such as ionic or hydrogen bonding. Strength of such bonding falls in the range of ~250 to 20 kJ/mol. Earlier reviews concerning this type of assembling components within the flexible spacer or mesogenic group are available [64,65]. Recently this strategy is developing as the architecture of SCLCPs that can be very much broadened (Figure 9a) allowing at the same time for simpler synthetic methods. In general, the self-assembly can occur for polymer or monomer, which is later polymerized in a subsequent step (Figure 9b) [66].

**Figure 10:**
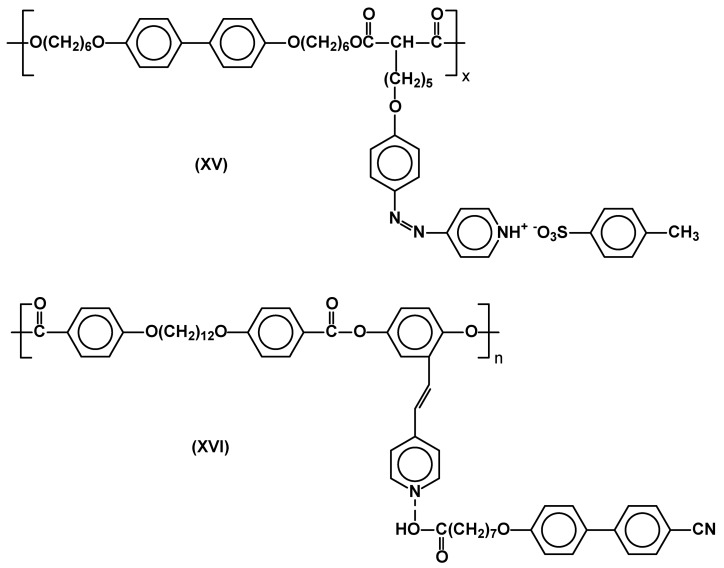
Self-assembled main/side chain poly(esters).

Novel SCLC polysiloxanes use the interaction of carboxyl terminated flexible spacer with stilbazole [34] or imidazole [35] moieties of rod-like mesogens. Bent-core side chain polymethacrylates based on the same type of hydrogen bonding (regarded as weak) were described. Some of them exhibit stable smectic phases up to over 200°C [16]. Synthesis of main-chain/side chain polymers, in which the structure of pendant side groups involved ionic (**XV**) or hydrogen bonding (**XVI**) has been reported [65] (Figure 10). Photorefractivity of SCLCP’s, which possess a hydrogen bonded moiety was compared to that of analogues with no hydrogen bonding group, but the same mesogen of nitrobezylideneaniline type. The hydrogen-bonding polymer (via methacrylic acid monomeric units) was found to exhibit large diffraction efficiencies below the glass transition temperature [67].

## 4. Novel side chain and main/side chain polymer structures

Although, in the last century hundreds of structures of side chain LC polymers have been synthesized, there are still many new reports dealing with various novel architectures. In general they can be divided into:

- polymers with new type of side chains designed usually for special applications (i.e. ferroelectrics, switchable photo-electric systems, materials with exceptionally wide or unusual temperature ranges of mesophase, self-assembling systems, ionic systems)
- polymers with new types of main chains or main/side chain systems, showing unexpectedly good (or sometimes weak) liquid crystalline properties due to side chain/main chain interactions.

Below, selected new structures are presented, representative for current main areas of research in the field.

### 4.1. Novel side chain polymer structures

In recent years, growing attention was devoted to studies of side-chain liquid-crystalline polymers with ionic groups (SCLCIs). The existence of ionic groups gives SCLCIs wider ranges of properties and extends their application fields. Side-chain liquid-crystalline ionomers play an important role in search for biocompatible materials as well as models for understanding complex biosystems [64].

**Figure 11:**
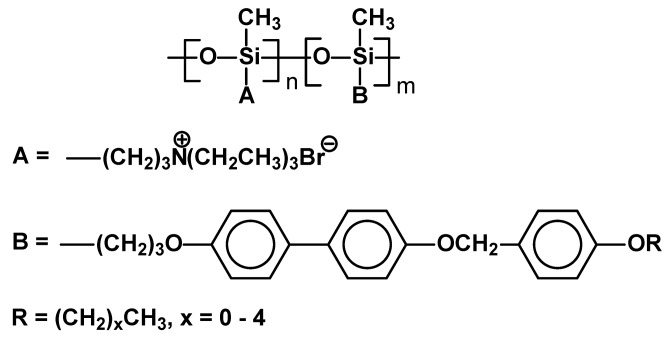
Ionic SCLCP based on commercial poly(methylhydrosiloxane).

Commercial polymethylhydrosiloxane [68] with M_W_=700–800 was used in a typical hydrosilylation of 4-(4-alkoxybenzyloxy)-4’-allyloxybiphenyls or mixtures of allyl triethylammonium bromide and 4-(4-alkoxybenzyloxy)-4’-allyloxybiphenyls, which produced several SCLC copolymers with (and without) ionic groups (Figure 11). It was found that non-ionic SC polymers exhibited smectic and nematic mesophase (for example: Cr 129 Sm 152 N 190 I) and after addition around 46-50% of ionic groups they generated only smectic phase, usually in a relatively narrow temperature range (Cr 164 Sm 181 I). Despite X-ray studies the type of smectic phase organization was not revealed.

Cholesterol derivatives as components of side chains are still of significant interest. Such systems usually generate chiral nematic and smectic phases which were found to be useful in many electro- and thermooptical applications [69].

**Figure 12:**
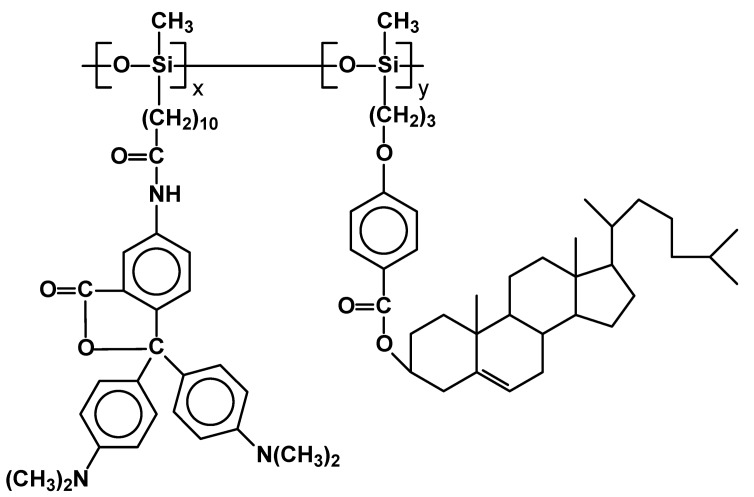
Structure of copolysiloxanes with cholesteric and malachite green lactone moieties in side chains.

New side-chain polysiloxanes containing cholesteryl 4-allyloxybenzoate and malachite green lactone 5-(undec-10-en-1-ylamidate) were synthesized as potential material for photochromic systems (Figure 12). They were based on polymethylsiloxane chain of molecular weight M_n_ = 800-900. Depending on x and y they generate SmC phase in the range of 62–278 °C. However, they do not exhibit any cholesteric phase, despite containing from 75 to 90% of monomeric units (m.u.) bearing cholesteric derivatives in side chains. Wide temperature ranges of SmC phase place them as potentially useful photochromic materials. [70]

**Figure 13:**
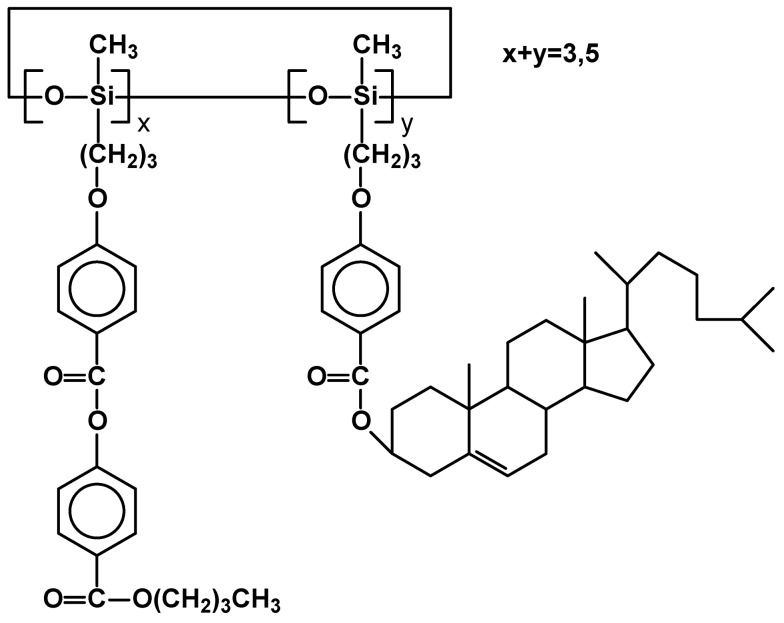
Structure of cyclosiloxanes with cholesteric and benzoxybenzoate mesogenic units.

In order to obtain polymers with a broad temperature range of blue phase and to study the role of chiral component cholest-5-en-3-yl(*3b*)-4-(2-propenyloxy)-benzoate and butyl-4-[4-(2-propenyloxy)-benzoxy]benzoate mesogens were grafted on cyclosiloxanes [71]. Depending on proportion of m.u. x and y (Figure 13) their LC properties slightly changed. Except for homopolymer, all others generated both N* and blue phase. The most stable blue phase was found for material with 2% of y m.u. (T_m_ 121 N* 196 Blue phase 224 I ) and the most narrow for the one having 8% of y m.u. (T_m_ 119 N* 195 Blue phase 214 I). The mesophase temperature ranges of the blue phases of the polymers are as broad as 20°C, significantly improving properties of material reported previously [69]. 

**Figure 14:**
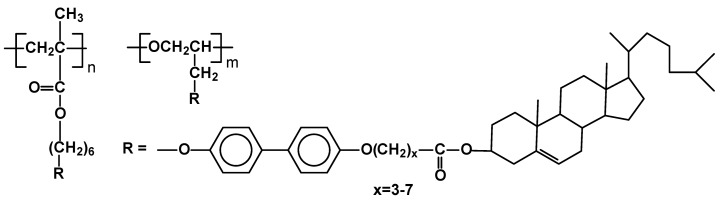
Poly(acrylates) and poly(epoxides) having two mesogenic moieties in the same side chain.

The synthesis of two types of side-chain liquid crystal polymers, having two mesogenic moieties in the same side-chain, of which one is a chiral nematic cholesterol moiety was published (Figure 14) [72]. One had polymethacrylate and the other polyepoxide main chain. Polymethacrylates generated only SmA phase up to isotropization at 208-285°C, depending on the length of (CH_2_)_x_ spacer, separating cholesteric group from the second part of mesogen. All poly(epoxides) have shown SmA phase as well, but also another unclassified smectic phase, which occurred before isotropisation at temperature 144-233 °C.

**Figure 15:**
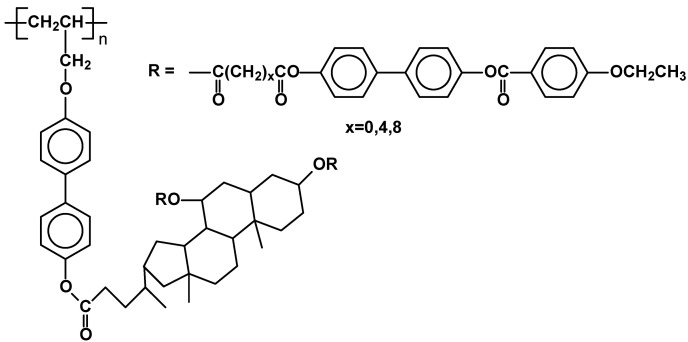
Polyolefin with side chains having chonediol moieties.

Steroids, such as chenodeoxycholic acid and cholesterol have similar molecular structures. In contrast to the extensive studies of side-chain liquid crystalline polymers containing cholesterol derivatives, very few cases of side-chain liquid crystalline polymers containing chenodiol were reported, most probably due to difficulty of their synthesis. The polymers have been recently obtained by free radical polymerization of terminal vinyl derivatives bearing chenodiol moieties [73]. The polymers exhibited very high phase transition temperatures, generating nematic mesophases over 200°C which underwent isotropisation at ~300°C, as proven by POM, DSC and X-ray studies (Figure 15).

During the 90s, ferroelectric LC have been extensively studied because of their short response time and memory effects toward an applied electric field [74]. These properties are expected for many electro-optical applications. Attempts to obtain polymer with stable SmC* phase, having good ferroelectric characteristics are still being continued.

**Figure 16:**
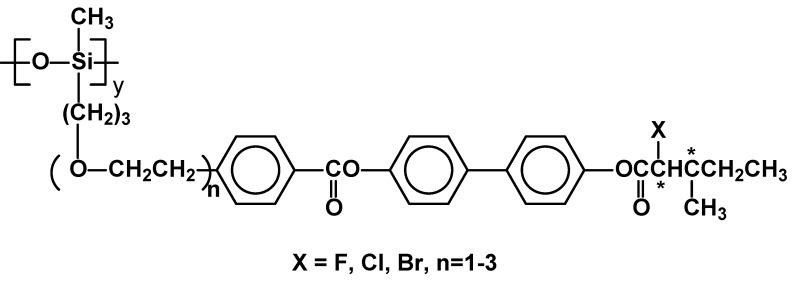
Polysiloxanes with chiral, halogenated mesogenic units and oligo(oxyethylene) spacers.

A new series of ferroelectric liquid crystal polysiloxanes with a halogenated chiral centre, oligooxyethylene spacers, and an ester core unit, containing three aromatic rings have been reported. (Figure 16) [75]. All of them exhibited the chiral smectic C phase (for example T_g_ 26 SmC* 139 Ch 160.5 I forX=Cl, n=3) except for the one containing bromine in the side chain. A wide SmC* temperature range was obtained and it expanded while the length of oxyethylene spacer increased.

**Figure 17:**
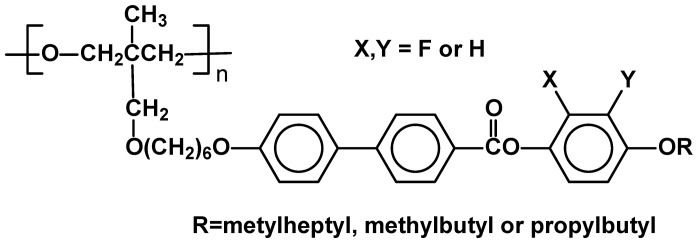
Poly(oxetanes) containing fluorophenyl moieties in their side chains.

It is already well established, that substitution of hydrogen atoms by fluorine leads to materials with wider temperature ranges of mesophase existence [76]. It is especially important for opto-electronic materials. Two series of side-chain liquid crystalline polymers were reported, having poly(ether) main chain and with 2- or 3-fluorophenyl moieties in chiral mesogen core (Figure 17). The number average molecular weight (M_n_) of resulting polymers were between 5,000 and 14,500 as measured by standard GPC. All the polymers exhibited expected SmC* as well as SmA phase, while two of them generated also tilted smectic phase, of unknown molecular organization, in very wide range of temperature (for example T_g_ -0.3 SmC* 123 SmA 137 I, T_g_ 19 SmC* 190 SmA 227 I). They are good film forming materials, which makes them excellent candidates for ferroelectric applications [37].

Azobenzene-containing polymers have been the subject of intensive research for photonic applications, *e.g*. such as laser beam addressed recording and holography. The azobenzene side chain moiety has a unique “push-pull” feature due to reversible trans-cis rearrangement on irradiation. The study over such the systems started in 90’s, however, they are still extensively investigated.

A series of poly{4’-{(X-methacryloyloxyalkylene)methylamino}-4-nitroazobenzene} liquid crystalline polymers (where X is the number of methylene groups in the spacer and varies from 2 to 12) have been recently studied. The polymers with molecular weight from 3,200 to 8,600, when stabilized in their glassy state, exhibited only smectic mesophase (except for X=2) in the range of 70-185°C up to 110-225°C depending on “X” and molecular weight. They decompose before reaching isotropisation temperature. The effects of pump irradiation of thin films of the polymers were studied proving that it is possible to create a stable anisotropy of the sample by photoinduction [77].

**Figure 18:**
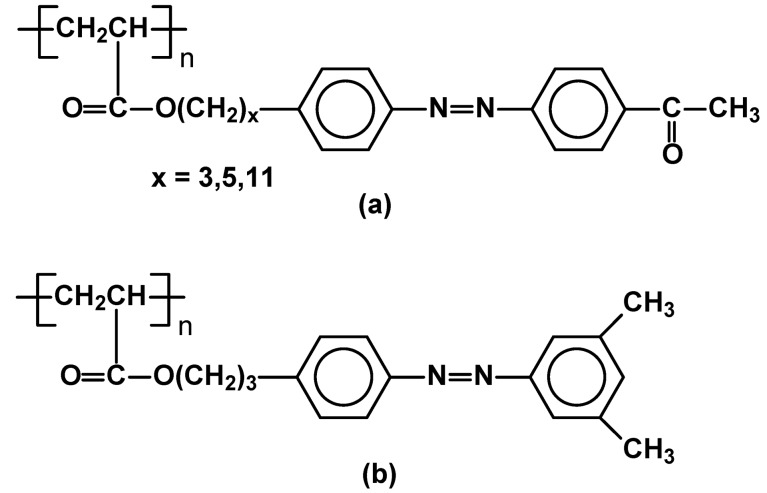
Structures of (a) Poly[ά-{4-[(4-acetylphenyl)azo]phenoxy}alkyloxy]acrylates, and (b) poly{3,5-dimethyl-{[4-(acryloyl-3-oxypropyloxy)phenyl]-azo}benzene}.

Another type of azobenzene side chain LC polymers was described. Poly[ά-{4-[(4-acetylphenyl)azo]phenoxy}alkyloxy]acrylates (“*a”* in Figure 18) were reported to exhibit nematic mesophase (Cr 108 N 272 I for n=3), when poly{3,5-dimethyl-{[4-(acryloyl-3-oxypropyloxy)phenyl]-azo}benzene} (*“b”* on Figure 18) generated smectic one (Cr 69 S 124 I) [78].

**Figure 19:**
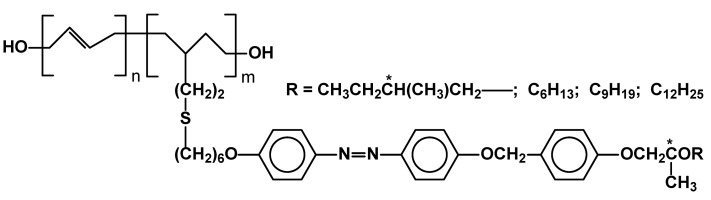
Polyolefins with thiol based azobenzyl side chains.

An unusual combination of SCLCPs bearing side groups with mesogenic thiol moieties and photosensitive N=N groups in the molecular core were also studied. Their smectic phase in the range of Cr 30-80 Sm 120-140 I depending on R group was though vaguely proved (Figure 19) [79].

**Figure 20:**
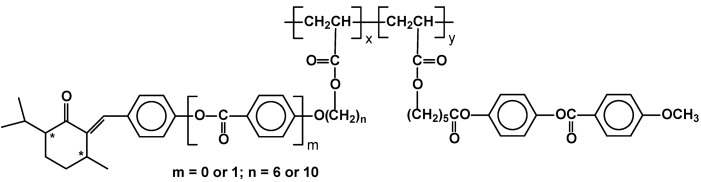
LC copoly(acrylates) with switchable arylidene-menthan-3-one (x) moieties in their side chains.

Another type of photochromic, switchable polymers were studied by the Shibaev group. A new family of combined chiral-photochromic copolymers is based on arylidene-menthan-3-one side chains capable of trans-cis isomerization on UV irradiation. (Figure 20) The combination of typical, smectic phase generating unit and chiral, trans-cis switchable units anchored on the same main polymeric backbone leads to materials undergoing controlled changes of their helical twist. It has been proved by detailed X-ray and photochromic studies that the light can be used as a very effective method to control local supramolecular structure and optical properties of the polymer thin films. The authors claimed that the polymers could be promising candidates as new materials for information storage, color data recording, color display technology, holography, and color projection systems [80].

So called mesogen-jacketed liquid crystalline polymers have been recently reviewed. They exhibit good film forming properties. The difference with traditional side chain polymers is that in this case mesogenic groups are laterally attached [40] (see also Section 2.3).

**Figure 21:**
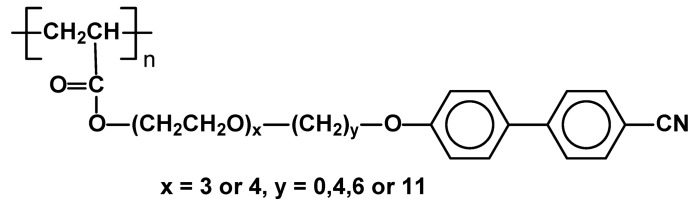
SCLC poly(acrylates) with cyanobiphenyl mesogenic units attached via oligo(ethylene oxide) and/or alkyl bridges.

A new type of side chain liquid crystal polymer with segmented spacers, consisting of cyanobiphenyl rigid moieties in an oligo(ethylene oxide) segment and an alkylene segment has been reported. Polymers of molecular weights 7,400-6,000 were made from relevant acrylic monomers. Most of those polymers with x=3–6 and y=4-11 exhibit SmA phases in relatively low temperatures (for example x=3, y=6 Cr -40 SmA 40 I) in comparison with analogous polymers with typical methylene bridges of similar length. (Figure 21) On the other hand polymers with spacers composed of oligo(ethylene oxide) units only (y=0) did not show mesomorphism at all. It seems that in the systems with long spacers, the spacer itself needs to be semirigid to form a material able of generating stable mesophases [81].

Apart from the main current areas of research in the field of SCLCPs, described above, there are also numerous publications about new structures which cannot be easily classified as ferroelectric, switchable, cholesteric or laterally attached systems.

**Figure 22:**
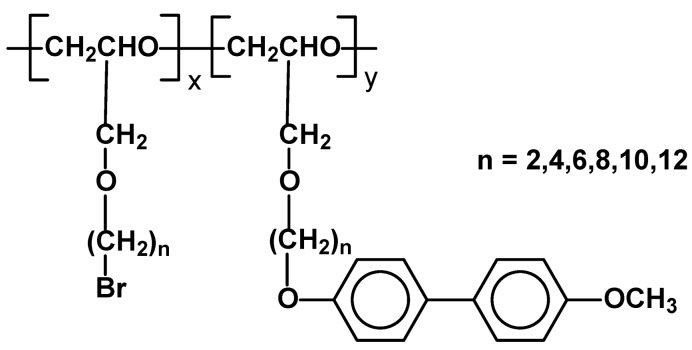
Poly(ω-bromoalkyl-1-glycidylether) with part of mers (y) substituted by 4’-methoxybiphenyl-4-oxy mesogenic groups.

High molecular weight (M_n_ = 150,000 – 200,000 D) side chain poly[ω-(4’-methoxybiphenyl-4-oxy)alkyl-1-glycidylether]s have been obtained by modification of poly(ω-bromoalkyl-1-glycidylether) (Figure 22)*.* Depending on “n” they exhibited typical smectic or nematic mesophases. The temperature range of mesophase formation were from Cr 17 SmC 119 (n=12) till Cr 99 N 143 (n=4) [82].

In a similar way analogous polyethers with cyanobiphenyl side groups have been obtained by the same research group. The polymers also exhibited nematic or smectic mesophases depending on “n” – Cr 17 SmA 127 I (for n=10) and Cr 37 N 92 I (for n=2) [83]. Similar polymers with alkoxybiphenyl mesogenic moieties, but bearing –CH_2_Cl groups instead of alkoxybromo ones have also been successfully synthesized. They generated various smectic and nematic mesophases, depending on number of methylene groups in spacers and on alkoxy end groups (from Cr 24 N 41 I to Cr 96 SmA 156 I) [84]. They can be synthesized by simple modification and in some cases exhibited wide temperature range of mesophase formation. The disadvantage is lack of precise control of the extent of modification and therefore lack of stable, reproducible properties.

**Figure 23:**
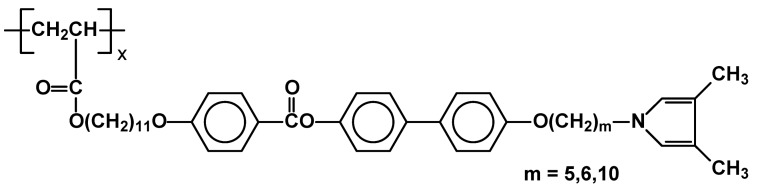
The structure of SCLCP’s with dimethylpyrrole moieties.

Polypyrrole and its derivatives have attracted attention because of their easy electrochemical or chemical oxidative polymerization, good environmental stability, and high conductivity in the oxidized state. The first example of side-chain liquid crystalline polymers containing intact 3,4-dimethylpyrrole moieties were prepared by free radical vinyl polymerization of bifunctional monomer N-{{ω-{4-[4’-(11-acryloyloxy)undecanoxybenzoyl]biphenyleneoxy}alkyl}}-3,4-dimethylpyrrole. (Figure 23) (M_n_ = 10,400-16,500). All the polymers showed nematic phase only, despite the fact that monomers exhibited complex LC behavior involving several intermediate smectic phases. Polymers with 10 methylene units between dimethylpyrrole moieties and the remaining part of the side chains (m=10) had the lowest phase transition temperature (Cr 92.5 N 176 I), while the polymer with five methylene units (m=5) had the highest ones (Cr 126 N 213.5 I) [85].

**Figure 24:**
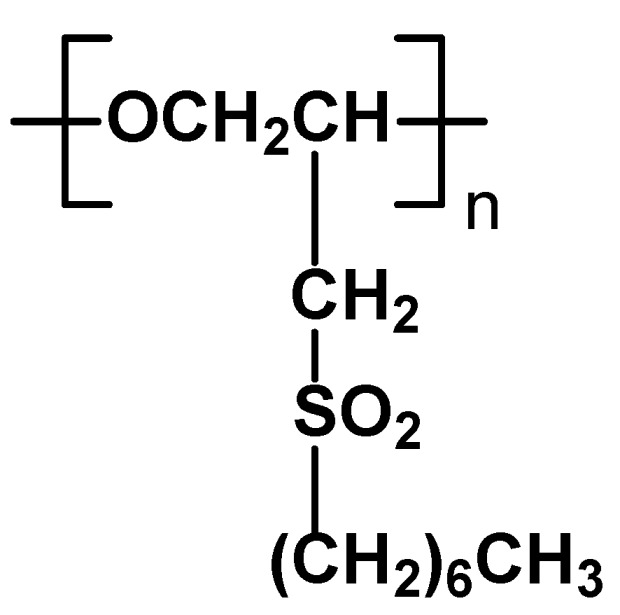
Polymers containing an oxyethylene backbone and n-octylsulfonylmethyl side groups able to exhibit tack effect.

Tack properties of polymers containing an oxyethylene backbone and *n*-octylsulfonylmethyl side groups have been described. Polymers with molecular weigh of 53,000-94,000 have been obtained by reaction of commercially available poly[oxy(chloromethyl)ethylene] with sodium *n*-octanethiolate under mild conditions and subsequent oxidation with *meta*-chloroperoxybenzoic acid (Figure 24). The polymers exhibit liquid crystalline properties despite the lack of typical rigid mesogenic units. Such the behavior has been already reported in the case of polysilanes and polysiloxanes [86]. They generate SmA phases at 80–160 °C as proved by DSC and X-ray studies. The ability of this polymer to form an ordered phase is attributed to the strong dipole-dipole interaction between the sulfone groups. Surface properties of one of the polymers were studied in details using NEXFAS and AFM techniques. Thermomechanical analysis proved that the polymer showed strong and reversible tack effect. It was found that surface properties of this polymer change dramatically at the transition temperature. Phase transition process modifies orientation of side-chain groups and main chain groups at the surface leading to dramatic changes of bulk viscoelastic properties, which results in switchable tack behavior over a narrow temperature range [87,88].

Liquid crystalline organization of disk-shaped mesogens results in the formation of various columnar phases which lead to unique optoelectronic properties important for many potential applications such as solar cells or field effect transistors [89]. Discotic liquid crystalline polymers have been much less explored than the calamitic ones due to limited number of available disc-like mesogens, which can be attached as side groups [90].

**Figure 25:**
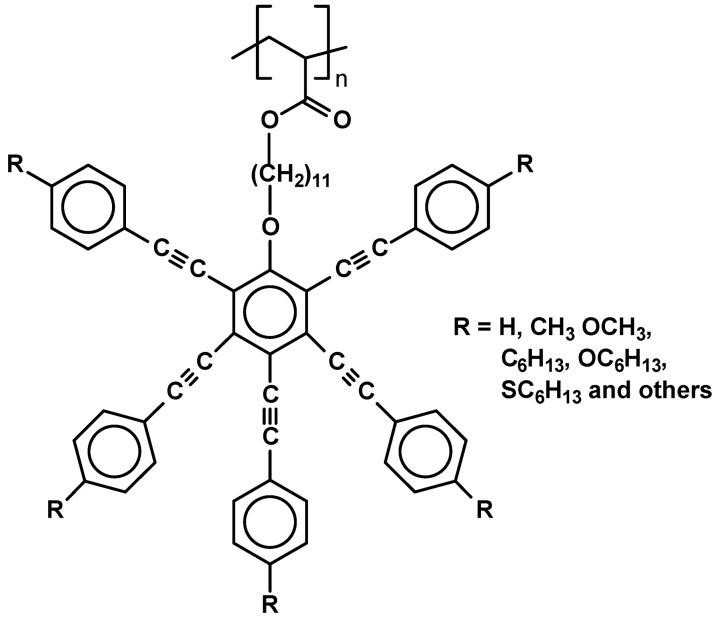
Polyacrylate bearing pentakis(phenylethynyl)phenoxy discotic mesogenic units.

One of the most interesting recent results obtained for discotic SCLCPs was a discovery that they are able to generate not only columnar phases but also so called “nematic discotic phase” (N_D_), once their flexible spacers are long enough. An example of the first polymeric system able to generate such the phase was published in 2000. It was made by modification of poly(acryloyl chloride) with relevant 11-[pentakis(phenylethynyl)phenoxy]undecan-1-ols (Figure 25). The resulting products of unknown molecular weight generated discotic columnar (N_col_) as well as nematic discotic mesophases (T_g_ 43 N_col_ 164 N_D_ 235 I for R=CH_3_ and T_g_ 41 N_D_ >200 for R=OCH_3_), as proven by DSC, POM and X-ray studies [91,92,93].

The polymer with R=OCH_3_ was doped with various low molecular charge transfer complexes which significantly changed its phase behavior leading to formation of N_col_ and lamellar discotic (N_L_) mesophases [92].

**Figure 26:**
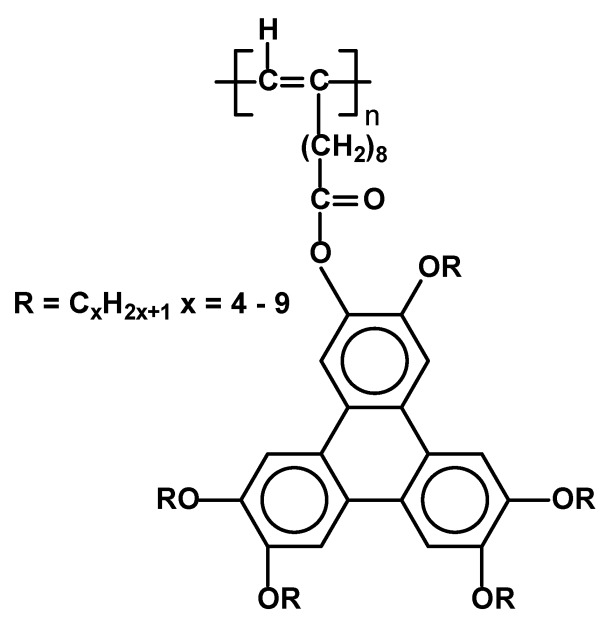
Poly(1-alkyne)s carrying triphenylene discogens.

Another type of SCLCPs (Figure 26) has been described recently [90]. The novelty of the system does not lie in side chain moieties (substituted triphenylenes), as similar ones have been attached to polyacrylate and polysiloxane backbones [89], but rather in a combination of rigid poly(alkene) main chains with discotic type mesogens, which according to authors should lead to materials with luminescent and photoconductive properties. The polymers were synthesized using organometallic catalysis in polymerization of triphenylene-containing 1-decynes, leading to expected substituted poly(alkenes) having M_w_ in the range of 16,000 – 20,000. All the resulting polymers exhibited highly organized columnar hexagonal mesophase at temperatures over 100 °C ( T_g_ 113 Col_h_ 156 I for x=5; T_g_ 116 Col_h_ 139 I for x=9) as proved by DSC, POM and X-ray studies. 

### 4.2. New side/main chain polymer systems

Until 1995 a vast majority of possible, typical architectures of polymeric chains have been already applied as SCLCPs’ main backbones. This includes poly(acrylates), metacrylates, poly(siloxanes), poly(ethers), poly(esters), poly(amides), etc. [94,95]. However, in the case of main chain/side chain polymeric systems, the structure-property relationship becomes an important issue. Better understanding of main chain – side chain interaction are of primary importance. Among others, effect of stereoregularity, density of mesogens along main chain and in side chains as well as influence of rigidity of main backbones is being studied.

**Figure 27:**
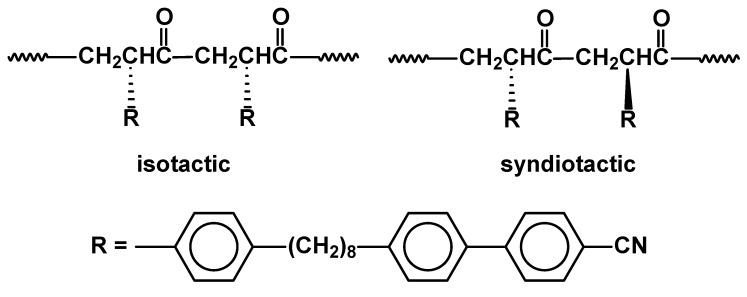
Isotactic and syndiotactic poly(ketones) bearing cyanobiphenyl mesogenic groups.

Stereoregular, isotactic and syndiotactic poly(ketones) have been prepared by alternating copolymerization of cyanobophenyl derivatives of styrene with carbon monoxide (Figure 27). The reactions were carried out in the presence of various chiral palladium organometallic complexes (Pd(II)– (R,S)-BINAPHOS and Pd(II)–2,2-bipyridine) and led to the polymers with M_n_ = 9,000 and 14,000. Isotactic polymer did not exhibit any mesomorphism, whereas the syndiotactic one generated SmB phase at 28–123 °C and SmA at 123–157 °C [96]. Similar copolymers with 1,2-di(trifluoromethane)phenyl substituted side chains were obtained and the effect was found to be analogous [97].

**Figure 28:**
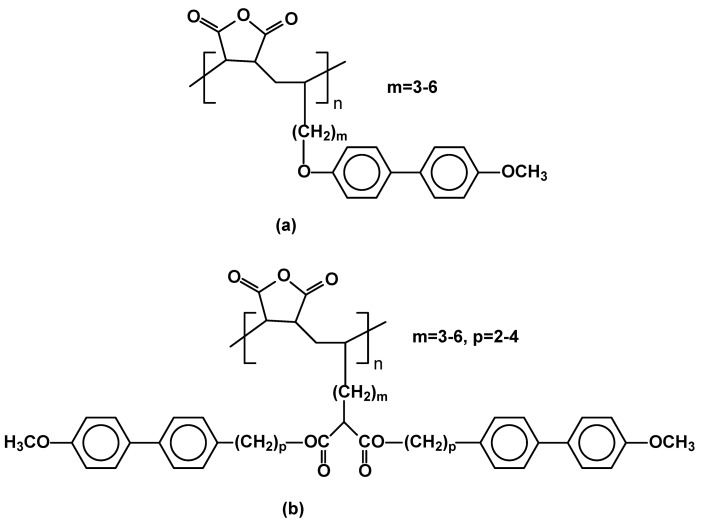
Maleic anhydride based polymers possessing (a) single methoxybiphenyl mesogenic moieties (b) double methoxybiphenyl side groups.

Side-chain liquid-crystalline polymers obtained from maleic anhydride (MA) and 1-alkenes containing methoxybiphenyl mesogens exhibit smectic mesophases, however, at relatively high and narrow temperature range (for example for m=6 in Figure 28a: Cr 129 SmB_hex_ 140 SmA_d_ 161 I). It was presumably due to the relative rigidity of their main chains and low density of mesogenic units along their main chains. They were obtained via free radical copolymerization of promesogenic, terminal alkenes with maleic anhydride, which lead to well defined polymers with degree of polymerization 11-26. In order to improve their LC properties, copolymers with doubled mesogen density were obtained by the alternating copolymerization of maleic anhydride with swallow-tailed 1-alkenes bearing two mesogenic units (Figure 28b). Due to the bulkiness of monomers a relatively low degree of polymerization has been achieved (8-11). The polymers exhibited slightly wider, than monomers, mesophase temperature range (for example for p=4, m=6: Cr 115 SmH_d_ 119 SmE_d_ 133 I). Doubling the mesogen density results in a higher degree of order of the mesophase - polymers with double mesogen have a tendency to generate SmH, SmE and SmA mesophases, whereas analogous single-mesogen polymers exhibit SmB_hex_ and SmA mesophases [98,99].

**Figure 29:**
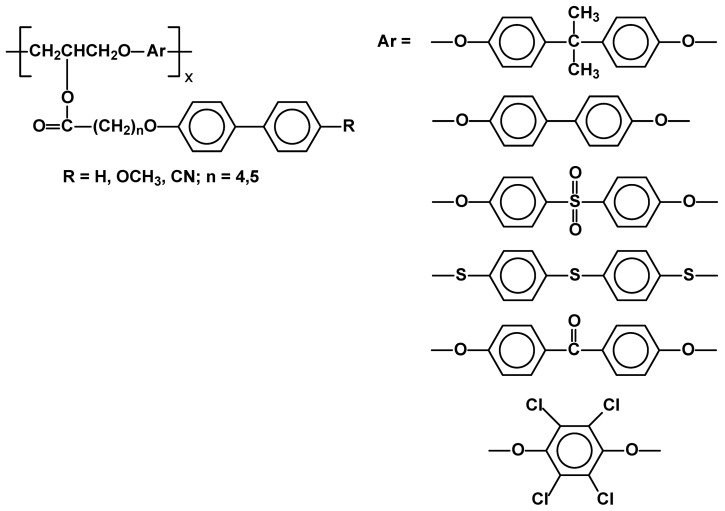
Main chain/side chain LC polymers obtained by reaction between epoxide and phenol esters.

A new method for synthesis of side-chain LCPs via reaction between epoxide and phenol esters, bearing mesogenic units (bisphenyl sulfone and bisphenyl isopropane alternatively separated by 1,3-propyleneglycol), makes it possible to vary the main-chain structure with a great ease (Figure 29). Tetrabutylphosphonium chloride (TBPC) and tetraphenylphosphonium chloride (TPPC) were used as catalysts in these polymerizations. Molecular weight of the products varied from 7,600 to 22,900. In order to get an appropriate conversion of monomers a relatively high temperature (130 °C–170 °C) and long reaction time (up to 72 hrs.) had to be applied. POM and DSC studies proved that the introduction of a kinked group into the main chain inhibited formation of mesomorphic phase in side-chain LCP’s. Only the polymer with bulky, linear aromatic structures in both main chain and side chains, showed liquid crystallinity in the temperature range of 60-110 °C of an unrecognized nature [100].

**Figure 30:**
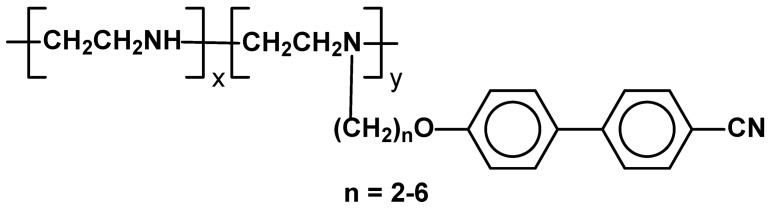
SCLCPs with poly(ethyleneimine)s (PEI) main chains and cyanobiphenyl side chains.

The use of poly(ethyleneimine)s (PEI) as a main chain appears interesting because the backbone with amine function offers many possibilities for chemical modification. Alkylation and quaternization allows for attachment of various bulky, ionic or interactive groups to the backbone generating attractive covalent or ionic SCLCPs. A series of PEI-based SCLCPs substituted with different number of cyanobiphenyl as pendant mesogenic groups, in which the spacer length varies between two and six methylene units, has been synthesized, by modification of commercially available PEI with bromo-functionalized mesogens (Figure 30). Due to equilibrium character of the reaction between bromides and imines, 100% of alkylation cannot be achieved (only up to 86% of the m.u. could be modified in this way). Almost all the obtained polymers exhibited nematic phase at temperature range depending on the number of methylene units in side chain spacers and proportions of modified and unmodified m.u.’s. Broad temperature range of nematic phase existence was obtained for polymer with the shortest methylene bridge (n=2) and 86% of alkylation (Cr 33 N 168 I). 69% of substitution seems to be a required minimum for mesomorphic phase formation [101].

**Figure 31:**
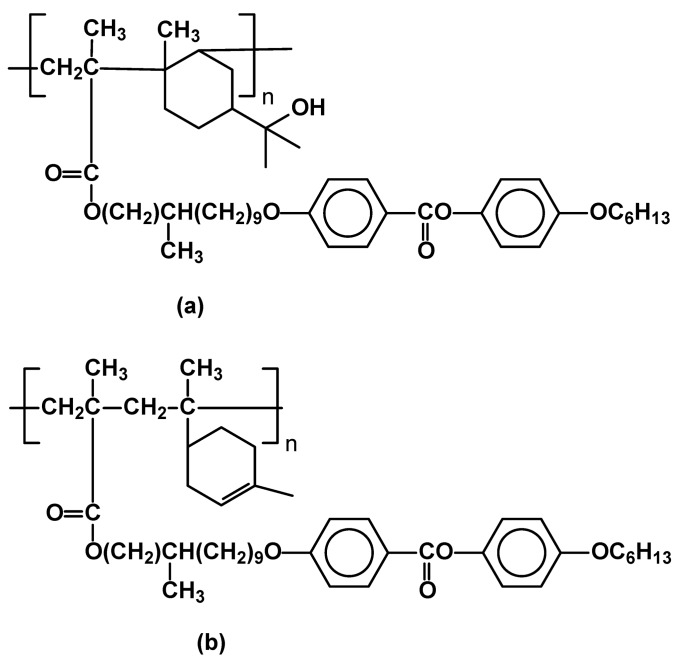
Side chain alternating copolymers based on (a) [α-terpineol-co-MMA] and (b) [limonene-co-MMA] main chains.

Novel SCLCPs, having [α-terpineol-co-MMA] main chains with a phenyl benzoate type mesogenic groups and polymethylene spacers, have been recently reported (Figure 31a). A comparison of thermal and liquid crystalline properties of polymethacrylates and poly[α-terpineol-co-MMA] based SCLCP’s has shown almost similar thermal behavior with formation of a different mesophase, though. For example, the phase transitions of liquid crystalline polyacrylate [102] are as follows: T_g_ 47 SmA 115 I, while for the analogous mesogenic group, the phase transitions of substituted [α-terpineol-co-MMA] are: T_g_ 52 N 120 I [21]. Similar temperature transitions exhibit also analogous [limonene-co-MMA] (Figure 29b) side chain polymers (T_g_ 48 N 119 I), however, they generate nematic mesophase instead of the smectic one, with the same mesogenic units [22].

## 5. Conclusions

Research concerning side chain liquid crystalline polymers (SCLCPs) is still a rapidly developing field due to many already existing and potential applications. In 2000-2008 more than one hundred new polymeric structures were described, exploiting extensive fundamental studies from the past. Variety of new systems can now be obtained as a result of large progress in synthetic methods. Tailored polymers can be much easier made using living and controlled polymerization techniques. RAFT and ATRP methods of polymerization have been successfully adopted to bulky monomers containing various mesogenic moieties. New catalysts and significant progress in studies of ring opening polymerization (ROP) and ring opening metathesis polymerization (ROMP) allow for much better defined polymeric structures.

An important input can be expected from research devoted to non-covalent side chain systems. Many efforts have been undertaken to obtain SCLCP’s layered nanomaterials by ion and hydrogen bonding driven self-assembly. It offers easier and more efficient synthetic approach to the whole spectrum of new polymers. Several new main chain/side chain structures have been synthesized throwing more new light on the influence on the structure-properties relationships. Especially laterally attached, chiral ferroelectric and chiral nematic systems were investigated in order to make new materials for various opto-electronic applications and nanotechnology. The current trend of “synthesis of properties”, not just only new structures is evident.
